# Gene expression in cardiac tissues from infants with idiopathic conotruncal defects

**DOI:** 10.1186/1755-8794-4-1

**Published:** 2011-01-05

**Authors:** Douglas C Bittel, Merlin G Butler, Nataliya Kibiryeva, Jennifer A Marshall, Jie Chen, Gary K Lofland, James E O'Brien

**Affiliations:** 1 Section of Medical Genetics and Molecular Medicine, Children's Mercy Hospitals and Clinics and University of Missouri-Kansas City School of Medicine, Kansas City, MO, USA; 2 Departments of Psychiatry & Behavioral Sciences and Pediatrics, University of Kansas, Medical Center, Kansas City, KS, USA; 3 Section of Cardiovascular and Thoracic Surgery, Children's Mercy Hospitals and Clinics and University of Missouri-Kansas City School of Medicine, Kansas City, MO, USA; 4 Department of Mathematics and Statistics, University of Missouri-Kansas City, Kansas City, MO, USA

## Abstract

**Background:**

Tetralogy of Fallot (TOF) is the most commonly observed conotruncal congenital heart defect. Treatment of these patients has evolved dramatically in the last few decades, yet a genetic explanation is lacking for the failure of cardiac development for the majority of children with TOF. Our goal was to perform genome wide analyses and characterize expression patterns in cardiovascular tissue (right ventricle, pulmonary valve and pulmonary artery) obtained at the time of reconstructive surgery from 19 children with tetralogy of Fallot.

**Methods:**

We employed genome wide gene expression microarrays to characterize cardiovascular tissue (right ventricle, pulmonary valve and pulmonary artery) obtained at the time of reconstructive surgery from 19 children with TOF (16 idiopathic and three with 22q11.2 deletions) and compared gene expression patterns to normally developing subjects.

**Results:**

We detected a signal from approximately 26,000 probes reflecting expression from about half of all genes, ranging from 35% to 49% of array probes in the three tissues. More than 1,000 genes had a 2-fold change in expression in the right ventricle (RV) of children with TOF as compared to the RV from matched control infants. Most of these genes were involved in compensatory functions (e.g., hypertrophy, cardiac fibrosis and cardiac dilation). However, two canonical pathways involved in spatial and temporal cell differentiation (WNT, *p *= 0.017 and Notch, *p *= 0.003) appeared to be generally suppressed.

**Conclusions:**

The suppression of developmental networks may represent a remnant of a broad malfunction of regulatory pathways leading to inaccurate boundary formation and improper structural development in the embryonic heart. We suggest that small tissue specific genomic and/or epigenetic fluctuations could be cumulative, leading to regulatory network disruption and failure of proper cardiac development.

## Background

The heart is the first major internal organ to form during embryogenesis, and its circulatory function is critical early on for the viability of the embryo. The development of integrated cardiovascular tissue is the result of multiple cell to cell interactions involving temporal and spatial events under genetic control. Failure of proper cellular differentiation, migration and apoptosis results in congenital heart disease (CHD), which is a major cause of childhood morbidity and death and remains a substantial challenge even in countries with advanced health care systems. The incidence of CHD is approximately eight per 1000 live births [1] making CHD the most common birth defect. Mendelian and chromosomal syndromes account for about 20% of all cases of CHD. The genetic mechanisms underlying non-chromosomal or non-Mendelian "sporadic" CHD, which account for the remaining 80%, are poorly understood. A multitude of genes and genetic networks contribute to the spatial and temporal specification of cell lineage required for proper embryological heart formation [2]. The molecular genetic components contributing to idiopathic CHD may include accumulation of multiple rare genomic and epigenetic variants converging to dysregulate cardiac developmental genes leading to mutational loading of developmental networks [3]. Although mutational loading is a credible explanation for the genetic etiology of CHD, there is a paucity of direct evidence supporting this proposition. Clearly a better understanding of the molecular genetic contributions to CHD is needed.

Tetralogy of Fallot (TOF) is a type of conotruncal congenital heart defect with an incidence estimated at five to seven per 10,000 live births, thus representing 5-7% of all congenital heart lesions. The occurrence of congenital cardiac lesions in the offspring of mothers with tetralogy of Fallot is approximately 3.1% [4-6] supporting a genetic contribution. TOF is characterized by a malalignment of the conal septum leading to a rightward deviation of the aorta. This results in a large ventricular septal defect and varying degrees of right ventricular outflow tract narrowing. There is variability in this patient population in the response to pulmonary artery growth and right ventricular function. Characterization of underlying aberrant gene expression leading to a better understanding of factors involved in the varied outcomes in children with conotruncal defects, will lead to a more tailored treatment strategy and improved outcomes.

Studies of the role of individual genes in human cardiac disease, as well as experimentation using animal models have dramatically improved our understanding of cardiac development. However, sporadic (nonmendelian, nonchromosomal) CHD, which account for 80% of all CHD, poses a challenge to scientific investigation. Epidemiological studies demonstrate an increased risk of CHD in siblings and offspring of individuals with sporadic CHD indicating a contribution of genes and/or shared environment [7]. These sporadic events are most often inherited from unaffected parents indicating incomplete penetrance [8]. Variable penetrance can be explained by differences in the genetic buffering capacity between individuals [9,10]. De novo events including sequence alteration or copy number changes can impact gene function or alter dosage and contribute to mutational load. Recessive mutations, if homozygous, may further destabilize regulatory networks.

Phenotypic stability is maintained by the ability of interconnected regulatory networks to compensate for both environmental and genetic variation. Mutational load can be compensated for through feedback mechanisms that control flux through regulatory pathways thus maintaining adequate residual function [9-13]. This concept implies that if threshold levels of flux are exceeded, compensatory mechanisms may fail, leading to inadequate development. Genetic studies of mutational loading of cardiac developmental pathways in the mouse model suggest limits to the buffering capacity of regulatory networks. When exceeded, the result is inadequate heart development. Furthermore, compound mutations produce phenotypes revealing the interconnected nature of members of cardiac developmental networks (reviewed by Bentham and Bhattacharya [3]). It is reasonable to expect homologous genes to behave similarly in humans implying that mutational loading could exceed buffering capacity leading to improper heart development.

Previously, five patients with TOF were compared to patients with RV hypertrophy, and 88 genes were identified with significantly altered expression in TOF but not altered in RV hypertrophy [14]. The authors listed potentially important genes with altered expression including *SNIP, A2BP1*, and *KIAA1437 *which were upregulated. *SNIP *interacts through the BMP signaling pathway which is essential for normal cardiac development. *A2BP1 *belongs to a novel gene family sharing RNA-binding motifs expressed in the developing embryological heart. *KIAA1437 *binds k-ras. K-ras deficient mice develop thin ventricular walls and die prematurely, suggesting a potentially important role for *KIAA137*in heart development. Genes markedly downregulated in TOF included *STK33, BRDG1*, and *TEKT2*. Sharma et al. examined four patients with TOF with a mean age of 0.8 years [15] and concluded that the upregulation of genes encoding *VEGF *and several extracellular matrix (ECM) proteins were the primary cause of TOF. Although the number of subjects examined was small, both studies suggested that altered gene expression in signaling pathways regulate heart development and contribute to TOF. We sought to examine gene expression variation in greater detail in tissues from children with TOF with a specific interest in determining if subtle changes in complex network behavior could lead to developmental irregularities.

We collected tissue samples (e.g., right ventricle, pulmonary artery and valve, thymus, pericardium) from a cohort of children with a similar congenital heart defect, tetralogy of Fallot. The surgical subjects included both children with known chromosomal abnormalities (22q11.2 deletion) and those with no known etiologies. For those subjects with no known etiology to explain TOF, we hypothesize that because of the similarity of the defect in the outflow tract, these children share a common developmental deficiency that may arise from different root causes, but underlying factors converge to disrupt proper temporal and spatial cell lineage specification leading to developmental deficiency.

## Materials

### Subjects

Our subjects were children less than two years of age with tetralogy of Fallot (TOF) requiring surgical reconstruction. Informed consent was obtained from a parent or legal guardian after reviewing the consent document and having their questions answered. All proper institutional review board approvals were obtained for this study. Our subjects included 16 nondysmorphic infants (eight male, eight female) with idiopathic TOF cardiac defects but without chromosome abnormalities (22q11.2 deletion). Three infants (one male, two female) with 22q11 deletion syndrome were also recruited for comparison of syndromic to nonsyndromic gene expression.

Comparison tissues from five (two male, three female) normally developing infants were obtained from LifeNet Health (http://www.lifenethealth.org, Virginia Beach, VA). LifeNet Health is a non-profit regenerative medicine company that provides bio-implants and organs for transplantation. The control subjects were matched for age to the study population and all control subjects expired due to non-cardiac related causes. LifeNet Health follows the following protocol for tissue recovery: 1) if the donor is placed in a refrigerated morgue within 12 hours of asystole, tissues can be recovered for up to 24 hours and placed in a 1-10°C sterile isotonic solution: or 2) if the donor is not refrigerated, tissues can be recovered for up to 15 hours and placed in a 1-10°C sterile isotonic solution. All donor tissue was de-identified, no donor confidential information was disclosed, and consent was obtained to use the tissue for research.

### Tissue

RNA was extracted from frozen tissues using PerfectPure RNA fibrous tissue Kit (5 Prime GmbH, Hamburg, Germany), according to the manufacturer's protocol. The control tissues (cryopreserved pulmonary homografts) were thawed per protocol in sterile conditions. One author (JEO) aseptically dissected samples from the control tissue from the right ventricle, pulmonary valve, and pulmonary artery matching what was done in surgery. All subjects enrolled in this study were undergoing surgical correction of TOF. Tissues samples obtained during patient surgery (attending surgeons JEO and GKL) were immediately de-identified and frozen. All tissue samples removed during surgery were excised by the performing surgeon for clinical indications utilizing standard of care procedures. While a subset of patients were previously palliated with a modified Blalock-Taussig (BT) shunt, the right ventricular outflow tract region from where the tissue was harvested had not undergone any previous surgical manipulation.

### Microarrays

Microarray data have been deposited at the Gene Expression Omnibus http://www.ncbi.nlm.nih.gov/geo/index.html. The accession number is GSE26125. The microarrays were CodeLink Human Whole Genome Bioarrays (Applied Microarrays Inc., Tempe, AZ) which contain >54,000 probes. The detailed microarray processing protocol can be found at http://www.appliedmicroarrays.com. Briefly, the poly (A) + RNA subpopulation (within the total RNA population) was primed for reverse transcription using an oligonucleotide containing a T7 RNA polymerase promoter 5' to an oligo d(T)24 sequence. After second-strand cDNA synthesis, the cDNA served as the template for an in vitro transcription (IVT) reaction to produce target cRNA. The IVT was performed in the presence of biotinylated nucleotides to label the target cRNA. This produced an approximate 1,000-fold to 5,000-fold linear amplification of the input mRNA.

A set of bacterial control mRNAs was included in the CodeLink™ Expression Assay Reagent Kit (Applied Microarrays, Tempe AZ) to act as controls for the cDNA synthesis and IVT reactions. These controls were added to the total RNA sample during target preparation. Each step of the CodeLink™ Expression Bioarray processing procedure, including target preparation and hybridization, was monitored using the control mRNAs. Additionally, bacterial control mRNAs can be used to estimate the sensitivity of RNA detection. Hybridization was performed overnight in a temperature-controlled shaking incubator with buffers supplied in the kit.

Post-hybridization processing included a stringent wash to remove unbound and non-specifically hybridized target molecules, a staining step with a Cy™5-Streptavidin conjugate, and several non-stringent washing steps to remove unbound conjugate. Following a final rinse, the microarrays were dried by centrifugation and scanned using an Agilent G2505B Microarray scanner (Agilent Inc., Santa Clara, CA). Analysis of the microarrays was performed with CodeLink Expression Analysis software (Applied Microarrays) and results were transferred to GeneSpring analytical software (Agilent Inc.) for further analysis.

Occasionally, signal intensities were recorded to be less than zero due to comparison of probe pixel intensity to the local background. Signal intensities cannot be less than 0, so the initial step in the analysis using GeneSpring version 7 software required that all values less than 0 be converted to a positive value, (i.e., 0.01) as done routinely [16,17]. This was a minimal intensity signal indicating that essentially no signal was detected. Normalization to the median baseline signal was accomplished using a global scaling process for all probe sets. Essentially, all microarrays were normalized to the median value of the control samples such that each measurement for each probe, in each microarray, was divided by the median of that probe's measurement in the corresponding control samples. Each measurement was then divided by the 50^th ^percentile of all measurements in that sample. This has the effect of minimizing discrepancies between an experiment and control baseline array value due to variation in preparation, hybridization or staining conditions or probe array lot number. The inclusion criteria for further analysis required a ''present'' or ''marginal'' signal in at least three of five control arrays, or three fourths of the arrays from the subjects with TOF. We chose to include only those probes that had a twofold difference between the TOF samples and controls due to the increased likelihood that these would achieve robust differences. Furthermore, within the TOF group, all values for the probe had to be in the same direction (up or down) relative to the control group. Differences between mean probe signal intensity were evaluated using a Welsh t-test without assuming equal variances and with a false discovery rate (fdr) of 5% or less using Bonferroni correction for multiple testing as undertaken in other studies [18,19].

We also examined the similarity in expression patterns by using the expression data from the right ventricle for detectable probes (~26,000) in an average linkage K-means clustering algorithm. This was performed using Pearson correlation as an expression similarity measure to allow clustering based on expression pattern relationships. Additionally, we were interested in identifying genes with similar patterns of expression across the three tissues, as they may have related functions. We therefore used expression values of all 2,932 genes with a significant change in expression in one of the three tissues and partitioned them into classes by K-means clustering. K-means clustering is a non-hierarchical, unsupervised, non-deterministic, and iterative approach to grouping genes with similar expression profiles into clusters. K-means clustering produces clusters of genes with a high degree of similarity within each cluster and a low degree of similarity between clusters so that the average behavior in each group is distinct from any of the other groups. The number of clusters, K, was set at nine.

We used Ingenuity Pathways analysis (IPA, Ingenuity Systems, Inc., Redwood City, CA.) for ontological assessment. IPA is a curated database and analytical bioinformatic system for identifying interactions, functions and interconnections (networks) between biological molecules. For those networks that appeared to have an excess of genes with reduced expression relative to the controls, we estimated the confidence interval of the proportion (i.e., is the proportion different than 50% or random) with an associated confidence level using statistical inference on the population proportion.

### QRT-PCR

To validate gene expression quantitative reverse transcription-PCR (RT-PCR) was performed on a subset of genes/transcripts using a QuantiTect SYBR Green RT-PCR kit (Qiagen, Valencia, CA) according to the manufacturer's directions and our previous experience [19]. The genes chosen were from the WNT or Notch pathway, with statistically significant differences in microarray expression data between the TOF and control groups in the microarrays data. Total RNA was isolated from tissues using a Perfect Pure fibrous tissue RNA extraction kit (5 Prime Inc., Gaithersburg, MD) according to the manufacturer's directions and quantified by spectroscopy. An equal quantity of total RNA (100 ng) from each subject, together with gene specific primers, were added to a reaction mix containing all components necessary for reverse transcription and PCR. The reaction was carried out in an ABI 7000 system (Applied Biosystems, Foster City, CA) beginning with a 30 min step at 50°C to allow for reverse transcription, followed by 15 min at 95°C. The intensity of the SYBR Green fluorescence was measured at the extension step of each of the 45 cycles of PCR The point at which the intensity level crossed the PCR cycle threshold (C_T_) was used to compare individual reactions. At least five replicates were performed on each sample for each gene. A dissociation curve was generated for all reactions, and reactions were run on agarose gels to verify the presence of a single band. Normalization of the Quantitative RT-PCR reactions used the 2^(-ΔΔC^_T_^) ^method with GAPD as the standardization gene for each sample to correct for minor experimental error as reported previously [18-21]. Normalized C_T _values were averaged to produce the mean C_T _value for each gene analyzed.

## Results

We obtained cardiovascular tissue (right ventricle, pulmonary valve and pulmonary artery) at the time of reconstructive surgery from 19 children with tetralogy of Fallot (16 idiopathic and three with 22q11.2 deletion syndrome) and employed whole genome gene expression microarray technology to characterize variation in gene expression patterns relative to tissues from five normally developing comparison subjects. As there was more than one probe per transcript, *probe *refers to an individual point on the array and *gene *refers to a single transcript. We detected the signal of approximately 26,000 probes on our microarrays (the signal strength for remaining probes was below the threshold of detection) representing about 50% of the genome using RNA isolated from the right ventricle (Table 1). Approximately 22,000 probes (41%) were detected in the valve and artery tissue perhaps reflecting less metabolic activity in these tissues producing fewer detectable active genes.

**Table 1:** Summary data

Sample	N	Number of genes detected in right **ventricle**^**1**^	Number genes **upregulated**^**2**^	Number genes **downregulated**^**2**^
Control	5	25265	na	na
TOF	16	20801	715	347
DiGeorge	3	18329	88	18

1. Passed filter

2. Statistically significant relative to controls

na = not applicable

We estimated inter-sample variability. The column vector B (Additional File 1, Table S1) contains the standard deviation for each sample. Each of the standard deviations can be interpreted as the inter-sample variability. The overall pooled sample standard deviation was 1.502 for all samples. In addition we estimated the generalized variance (GV) or determinant of the covariance matrix X. The GV value for X was 0.0023576.

Furthermore, we examined the expression of the genes in the 22q11.2 region, including *TBX1 *which did not have a detectable signal in any of the tissues examined. Other genes in the region (including *COMT, DGCR2, CRYBA4, ZNF74, FLJ36561, ARVCF, RUTBC2, GGT, MMP11*, Table 2) were unchanged in signal intensity in our subjects with idiopathic TOF. As expected, half the signal intensity was present in the three subjects with the 22q11.2 deletion supplying evidence of the reliable detection of gene expression levels in these tissues. We also validated the expression of three genes from the WNT (*DVL3 *and *WNT5B*) and Notch (*DTX3*) pathways with significant differences in expression between the TOF subjects and controls using quantitative reverse transcription qRT-PCR. All three had good agreement in expression levels detected by the two techniques (Table 3).

**Table 2:** Mean relative expression of genes1 on 22q11.2

Gene Name	Map	22q11.2 del	TOF	Control	RefSeq
COMT	22q11.21-q11.23	0.36 (0.04)	1.02 (0.15)	0.96 (0.09)	NM_000754; NM_007310
DGCR2	22q11.21	0.27 (0.10)	0.71 (0.23)	0.90 (0.25)	NM_005137
CRYBA4	22q11.2-q13.1	0.54 (006).	0.86 (0.32)	1.01 (0.10)	NM_001886
ZNF74	22q11.2	0.52 (0.01)	0.99 (0.12)	1.00 (0.15)	NM_003426
FLJ36561	22q11.23	0.40 (0.24)	1.18 (0.44)	1.00 (0.21)	NM_182520
CABIN1	22q11.23	0.52 (0.06)	0.53 (0.17)	0.99 (0.23)	NM_012295
ARVCF	22q11.21	0.57 (0.07)	1.01 (0.44)	0.92 (0.24)	NM_001670
FLJ14360	22q11.21	0.65 (0.08)	0.58 (0.17)	1.01 (0.13)	NM_032775
RUTBC2	22q11.23	0.69 (0.04)	0.92 (0.42)	0.94 (0.16)	
GGTLA1	22q11.23	0.58 (0.13)	0.61 (0.22)	0.98 (0.28)	NM_004121
DGCR8	22q11.2	0.54 (0.11)	0.59 (0.25)	0.99 (0.11)	NM_022720
GGT1	22q11.23	0.70 (0.36)	1.19 (0.32)	0.94 (0.26)	NM_005265; NM_013421; NM_013430
MMP11	22q11.2	0.37 (0.18)	0.77 (0.33)	1.00 (0.17)	NM_001145938
TBX1	22q11.21	Not detectable	Not detectable	Not detectable	NM_005992

1. Expression levels from microarray (fold change) relative to the normalized control values set at 1, mean (+/- sd).

**Table 3:** Comparison of QRT-PCR validation and microarray mean relative values1

Genbank	Gene Symbol	Map	Microarray	QRT-PCR	Pathway
NM_178502	DTX3	12q13.2	0.57 (0.22)	0.63 (0.30)	Notch
NM_004423	DVL3	3q27	0.38 (0.13)	0.53 (0.15)	WNT
NM_030775	WNT5B	12p13.3	0.53 (0.14)	0.15 (0.51)	WNT
NM_006172.3	NPPA	1p36.22	2.2 (0.59) (shunt) 19.4 (5.02) (no shunt)	2.1 (0.32) (shunt) 19.3 (0.82) (no shunt)	BMP

1. Expression level (fold change) relative to the normalized control values set at 1, mean (+/- sd).

**Figure 1:**
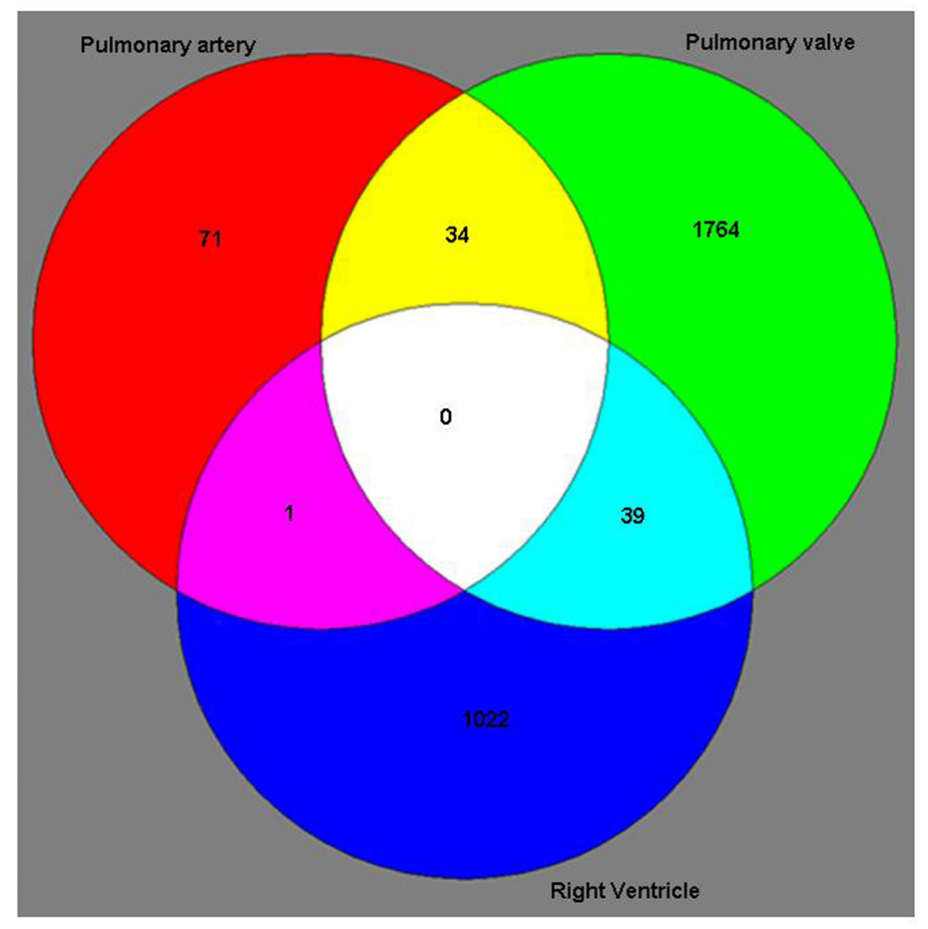
**Venn diagram showing the number of genes in each tissue with 2-fold changes in expression in the infants with TOF relative to controls**.

We identified 1,062 genes in the right ventricle which had a statistically significant change in expression of at least 2-fold in TOF subjects relative to our control tissue samples (see Figure 1). These 1,062 genes met a strict statistical threshold for significance: a false discovery rate (FDR) of 5% using Bonferroni correction for multiple testing. Likewise, our analysis identified 1,834 and 106 genes in the pulmonary valve and pulmonary artery, respectively with a 2-fold change in expression. The large number of genes with changed expression in the valve and ventricle relative to the artery may be a reflection of the primary defect originating in these tissues with secondary response in the artery. We compared the list of genes with a 2-fold change from the three tissues to identify genes in common (Figure 1). Interestingly, the ventricle and pulmonary valve had 39 genes in common, and the pulmonary valve and artery had 34 genes in common. However, there was only one gene with a 2-fold change in expression in common between the ventricle and the artery, and there were no genes with a 2-fold change in expression shared by all three tissues.

**Figure 2:**
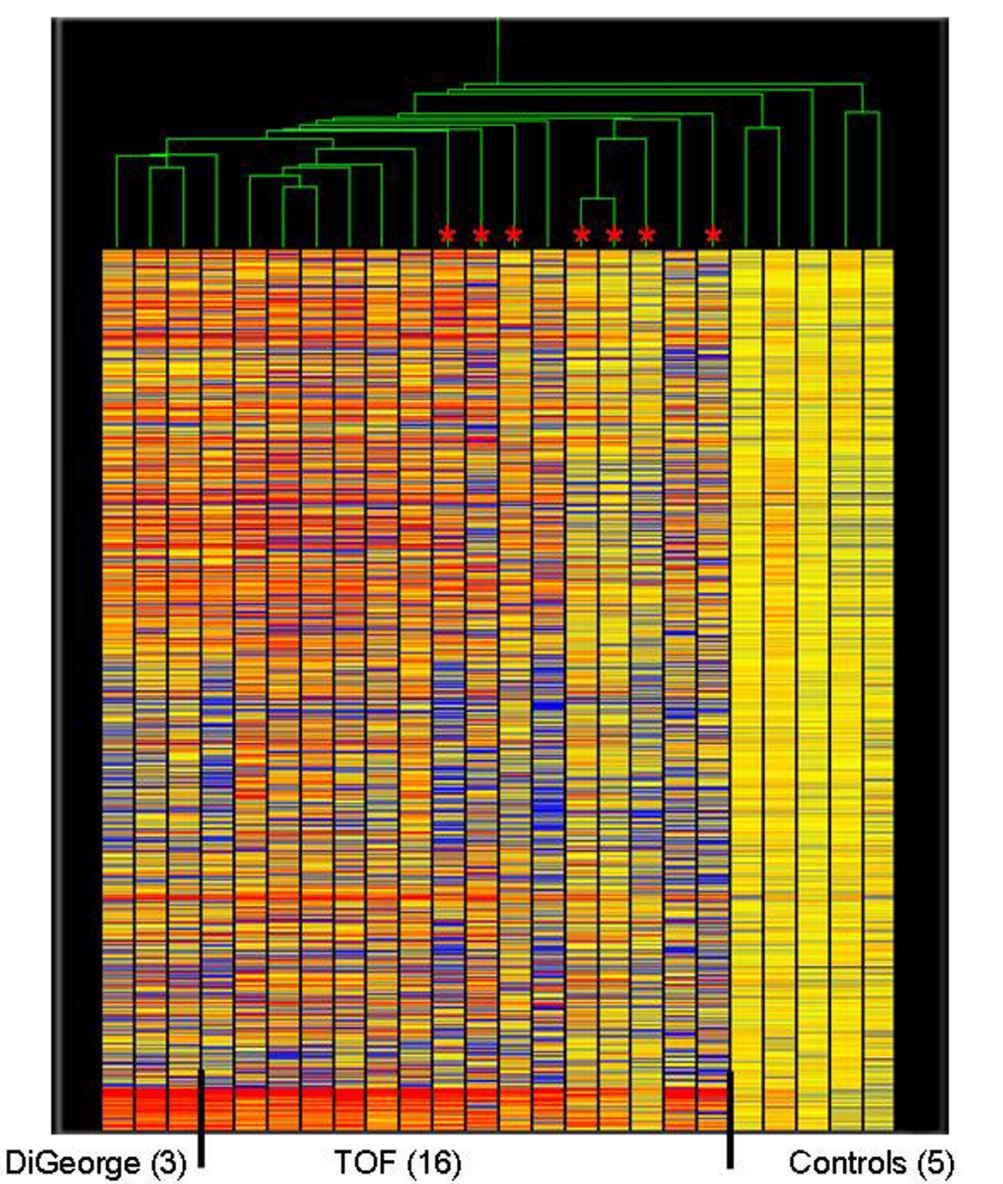
**Heat map with clustering based on all detectable genes in the right ventricle**. * Infants requiring an early shunt (i.e., more severe).

We used the expression data from the ventricle for detectable probes (~26,000) in an average linkage K-means clustering algorithm. The heat map (Figure 2) shows the gene tree resulting from the analysis. In Figure 2, five lanes on the right are from control samples and three lanes on the left are from patients with 22q11.2 deletion syndrome. The 16 subjects with TOF clearly are clustered together compared to control subjects, with the majority of the idiopathic subjects grouped separately from the 22q11.2 deletion subjects.

In an attempt to separate patients into differing levels of severity, we chose to group our patients based on those that required early intervention with a modified BT shunt placement (considered to be more severe), and those that did not require early intervention before complete surgical TOF correction. Within the group of TOF subjects, seven required a BT shunt placement early in life (red stars noted in Figure 2). These seven subjects were part of a group of nine patients that separate from the remaining TOF subjects. In general, a common pattern of global gene expression was seen in the TOF subjects differing from the controls, those subjects considered to have more severe conotruncal defects (those requiring early surgical intervention) tended to have distinct patterns of gene expression placing them on distinct branches of the tree.

We identified 645 genes which had a 2-fold change in expression between the severe and less severe groups with FDR at 5% and Bonferroni multiple test correction. The top canonical pathways identified included cardiomyocyte differentiation via BMP (bone morphogenetic protein). Two genes from this pathway, *NPPA *and *NPPB *(natriuretic peptide precursor A and B) were highly overexpressed in the TOF subjects relative to the controls, but were five times higher in the less severe TOF group relative to the early shunt group (Figure 2 shows a 3-fold and 15-fold change in less and more severe TOF subjects, respectively, relative to control subjects).

We have done ontological analysis of the 1,062 genes with altered expression in the right ventricle using Ingenuity Pathways Analysis software (Ingenuity Systems Inc., Redwood City, CA), as well as GeneSpring software and have identified several important relationships. The list of genes contained a number of heart development and function networks which were over represented. The top five biofunction networks identified were: *protein synthesis, cardiovascular disease, genetic disorder, neurological disease*, and *cell death*. The list of statistically significant canonical networks (Additional File 2, Table S2) included WNT and Notch pathways; each having several members with significantly altered expression.

Because of our interest in determining the expression patterns of other members of the WNT and Notch pathways, we used Ingenuity Pathways Analysis to visualize the expression levels regardless of statistical significance. [Additional files 3 and 4, Figures S1 and S2; green represents reduced expression (signal intensity) and red represents increased expression relative to the median of the controls and Additional files 5 and 6, Tables S3 and S4,]. Interestingly, expression of most members of the networks was reduced (green in the Additional files 3 and 4, Figures S1 and S2) in the myocardium of our patients with TOF, suggesting a pattern of mutational convergence leading to a general suppression of gene expression in the right ventricle of these developmentally important networks. As can be seen in Additional files 3 and 4, Figures S1 and S2, the expression levels in ventricular tissue for both networks were predominately downregulated relative to the controls. The number of downregulated genes in each network was significantly different from a discrete uniform distribution. This would be expected if the upregulated and downregulated genes were occurring by equal chance. For example, 67/109 (62%) of units (genes) were green in the WNT pathway (uncolored units were not counted as they were not detectable on the microarray, also see Additional file 5, Table S3 for values of probes). The true proportion of green units can be estimated to occur between 52% and 71%, with 95% confidence. Therefore, since a discrete uniform distribution would result in an equal proportion (50%) of green units (reduced expression relative to controls) to red (increased expression), and since the entire range of estimated values is greater than 50%, the number of green units occurs more often than expected by chance. A statistical test to determine if the proportion of green was different from 50% was performed resulting in a *p*-value of 0.017 indicating that the proportion of green was significantly different from 50%. Likewise, the analysis of the Notch pathway suggested that this pathway was also suppressed (18/22, 82% units are green with a *p*-value of 0.003, see Additional file 6, Table S4 for values of probes). Interestingly, the Notch pathway was also significantly suppressed in subjects with 22q11.2 deletions (*p *= 0.014), suggesting a common outcome resulting from different initiating parameters.

We attempted to evaluate if there was a general trend toward downregulation of gene expression in developmentally deficient tissues relative to normally developing tissue. First we analyzed the general distribution pattern of expression values in the TOF group and found no difference compared to the control subjects. Additionally, we examined several other pathways with comparable numbers of units (e.g., Caviolar mediated endocytosis [51 units], Oncostatin M signaling [22 units], tight junction signaling [106 units]) and found that they appear to have a discrete uniform distribution (i.e., an equal chance of reduced or increased expression relative to the control values, data not shown). Thus, it appears that the suppression of gene expression was confined to the WNT and Notch pathways which are known to regulate developmental patterns in the heart.

We were interested in identifying genes which had similar patterns of expression across the three tissues as they may have related functions. We therefore took expression values of all 2,932 genes with a significant change in expression and derived nine clusters of genes with similar expression patterns in the three tissues and imported the list of genes in each group into Ingenuity Pathways Analysis for ontological analysis. The resulting nine classes of genes ranged in size from 119 to 684 members (Additional file 7, Table S5). Within the broad category *Physiological System Development and Function*, cluster sets seven and eight had a statistically overrepresented group of genes involved in embryonic development. Cluster sets one, six, seven and nine all had significant numbers of genes involved in *Cardiovascular System Development and Function *suggesting that, in addition to having altered expression, many of these genes appear to have similar patterns of expression across the three tissues possibly signifying coordinated function. Interestingly, cluster set three had a significant group of genes from the Notch canonical pathway indicating that the members of the Notch pathway had similar expression patterns across these three tissues.

## Discussion

Congenital heart defects are the most common type of major birth defect, and account for the majority of morbidity and mortality related to birth defects. The origin of most congenital heart disease is thought to be multifactorial, implying contributions from anomalous gene expression and epigenetic factors, as well as environmental contributions.

Some of the genes involved in normal cardiogenesis include transcription factors (e.g., *NKX2.5, GATA-6, GATA-4, HAND1, HAND2*, and *NF-ATC*) which regulate the expression of genes in a tissue specific and quantitative manner, as well as soluble factors including bone morphogenic proteins (which acts as a positive facilitator of nodal induction and left-right asymmetry), transforming growth factor beta isoforms and fibroblast growth factor isoforms (these may play a role in cardiac hypoplasia) [22-24]. Many genes and networks identified in lower animals have similar roles in human cardiogenesis. Study of animal models has greatly advanced our understanding of the genetic mechanisms regulating heart development. In spite of the expanding knowledge of the genetic mechanisms involved in cardiac formation, there remain nearly 80% of children with congenital heart defects who do not have a known genetic defect.

A previous study of gene expression in cardiac tissues from a small number of children with TOF indicated changed expression of several genes of potential importance including *SNIP, A2BP1*, and *KIAA1437 *[14] which were upregulated, and genes markedly downregulated included *STK33, BRDG1*, and *TEKT*. In addition, another small study of gene expression in tissues from children with TOF concluded that gene expression changes in *VEGF *and altered levels of several ECM proteins were contributory to TOF. Our data indicated that the change of expression for these genes was in the same direction as previously reported, although only *A2BP1 *reached statistical significance in our analysis. Our sample size was larger with more strict correction procedures for multiple testing which may account for these genes not reaching the statistical threshold in our analysis.

Our data indicate that developmental deficiencies resulting in conotruncal defects are associated with distinct changes in gene expression. Many of these genes are involved in ameliorating the consequences of pulmonary atresia/stenosis. However, we identified a striking collective suppression of genes in the WNT and Notch pathways which are known to play critical roles in cardiac development. Genetic mechanisms which control embryonic heart formation are precisely regulated, both temporally and spatially. We suggest that the general downregulation of these pathways is an indication of faulty embryologic gene expression causing imperfect cardiac development in these children.

The *NPPA *and *NPPB *genes are tightly linked on human chromosome 1. During embryonic heart development, chamber myocardium is derived from the myocardium of the tubular heart and expression of the *NPPA *gene, activated via the BMP pathway, is one of the first hallmarks of heart chamber formation (OMIM, 108780). The *NPPA *and *NPPB *genes can also be activated by glucocorticoids and increased expression has been associated with hypertrophy. Thus the activation of these genes is associated with myocardial cellular proliferation. The reduced expression of these two genes in the group receiving an early shunt (considered to be more severe) compared to the group not receiving a shunt may be a biomarker for poorer conotruncal development and perhaps poorer prognosis.

The genes associated with clustered gene sets seven and eight, (Additional file 7, Table S5) containing genes involved in embryonic development may also be important for further study as they may represent altered pathways which may play a role in deficient spatial patterning as the fetal heart was developing.

As was seen previously [14,15], many of the differentially expressed genes were associated with compensation relative to the conotruncal anomaly. However, we believe we have identified a pattern of expression of developmentally important networks (e.g., WNT and Notch signaling networks) which supports the hypothesis that converging and accumulating factors disrupt regulatory networks controlling heart development during embryological development ultimately leading to tetralogy of Fallot. Moreover, an apparently similar suppression of the Notch pathway in children with 22q11.2 deletions (*p *= 0.014) suggests that factors leading to network suppression can arise from variable origins.

## Conclusions

The moderate suppression of two pathways which are important for heart development is consistent with the hypothesis that many rare nonsynonymous variants, each with a small impact (mutational loading), may accumulate and converge to dysregulate cardiac development. These variants may be a consequence of genetic (protein coding variants, promoter sequence changes or splicing variants) or epigenetic modification (e.g., methylation changes in CpG islands, histones or microRNA expression), which in summation could produce a shift in network function to impair cardiac development. Our observations suggest a more comprehensive genetic and genomic analysis should be undertaken to identify candidate disturbances influencing pathway integrity.

## Competing interests

The authors declare that they have no competing interests.

## Authors' contributions

DCB participated in study concept and design and coordination of the study, helped with the statistical analysis and drafted the manuscript. MGB participated in study concept and design, and helped to draft the manuscript. NK carried out the microarrays and the statistical analysis of arrays and helped to draft the manuscript. JAM participated in coordination of the study and helped to draft the manuscript. JC helped with the statistical analysis and helped to draft the manuscript. GKL participated in study concept and design, sample acquisition and helped to draft the manuscript. JEO participated in study concept and design, study coordination and sample acquisition and helped to draft the manuscript. All authors read and approved the final manuscript.

## Pre-publication history

The pre-publication history for this paper can be accessed here:

http://www.biomedcentral.com/1755-8794/4/1/prepub

## Supplementary Material

Additional file 1 **Table S1. Estimates of intersample variability**. Estimates of the variation between samples. Click here for file

Additional file 2 **Table S2. Canonical pathways associated with altered gene expression in TOF**. List of pathways associated with genes with changes expression in TOF relative to controls, p value and gene names. Identified using Ingenuity Pathways Analysis (IPA). Click here for file

Additional file 3 **Figure S1, WNT pathway**. WNT canonical pathway. Color corresponds to increase (red) or decrease (green) in signal intensity (expression) of genes in TOF subjects relative to control subjects (developed using the Ingenuity Pathways Analysis program). Click here for file

Additional file 4 **Figure S2, Notch pathway**. Notch canonical pathway. Color corresponds to increase (red) or decrease (green) in signal intensity (expression) of genes in TOF subjects relative to control subjects (developed using the Ingenuity Pathways Analysis program). Click here for file

Additional file 5 **Table S3. List of genes in the WNT pathway with fold change in TOF relative to controls**. Complete list of genes from the WNT pathway and the fold change in the right ventricle from subjects with TOF relative to control subjects. Click here for file

Additional file 6 **Table S4. List of genes in the Notch pathway with fold change in TOF relative to controls**. Complete list of genes from the Notch pathway and the fold change in the right ventricle from subjects with TOF relative to control subjects. Click here for file

Additional file 7 **Table S5. Ontological analysis of genes with a significant change of expression between subjects with TOF and controls**. Ontological analysis of clustered genes with a similar pattern of expression across the three tissues examined (right ventricle, pulmonary valve and pulmonary artery). Click here for file
