# Supplementary material for: Gene expression in cardiac tissues from infants with idiopathic conotruncal defects
Source: BMC Med Genomics. 2011 Jan 5;4:1. doi: 10.1186/1755-8794-4-1 (PMC3023653; doi:10.1186/1755-8794-4-1)
Supplement: Additional file 1 — Table S1. Estimates of intersample variability. Estimates of the variation between samples. [file 1755-8794-4-1-S1.DOC]

Supplemental Table 1. Estimates of intersample variability

| **GV** |
| --- |
| 0.0023576 |

| **Column vector B** | |
| --- | --- |
| Lab.021 | 1.7283345 |
| Lab.012 | 1.8021321 |
| Lab.022 | 1.3401455 |
| Lab020 | 1.5971694 |
| Lab018 | 2.2599115 |
| Lab007 | 1.9040746 |
| Lab010 | 1.9978013 |
| Lab025 | 1.7449699 |
| Lab024 | 1.6049611 |
| Lab016 | 1.7904971 |
| Lab009 | 1.8789279 |
| Lab017 | 1.9631047 |
| Lab015 | 1.794146 |
| Lab013 | 1.1633099 |
| Lab023 | 1.1193882 |
| Lab011 | 1.044945 |
| CONTROL9 | 0.4478013 |
| CONTROL8 | 0.3643501 |
| CONTROL7 | 0.5260874 |
| CONTROL5 | 0.3382957 |
| CONTROL4 | 0.4507327 |

|  |  |  |  |
| --- | --- | --- | --- |
