# Supplementary figures and images for: Gene expression in cardiac tissues from infants with idiopathic conotruncal defects
Source: BMC Med Genomics. 2011 Jan 5;4:1. doi: 10.1186/1755-8794-4-1 (PMC3023653; doi:10.1186/1755-8794-4-1)

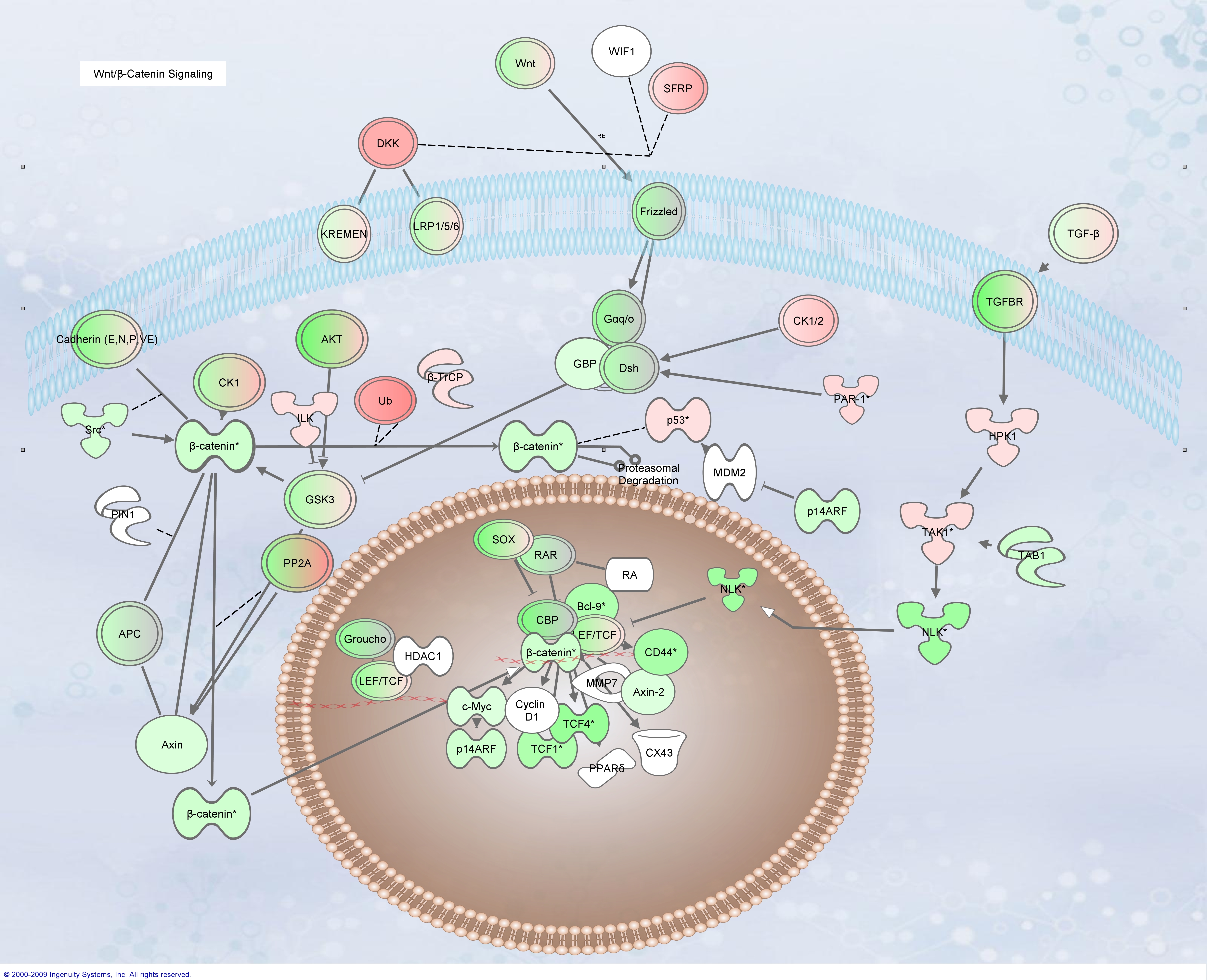

Supplement: Additional file 3 — Figure S1, WNT pathway. WNT canonical pathway. Color corresponds to increase (red) or decrease (green) in signal intensity (expression) of genes in TOF subjects relative to control subjects (developed using the Ingenuity Pathways Analysis program). [file 1755-8794-4-1-S3.JPEG]

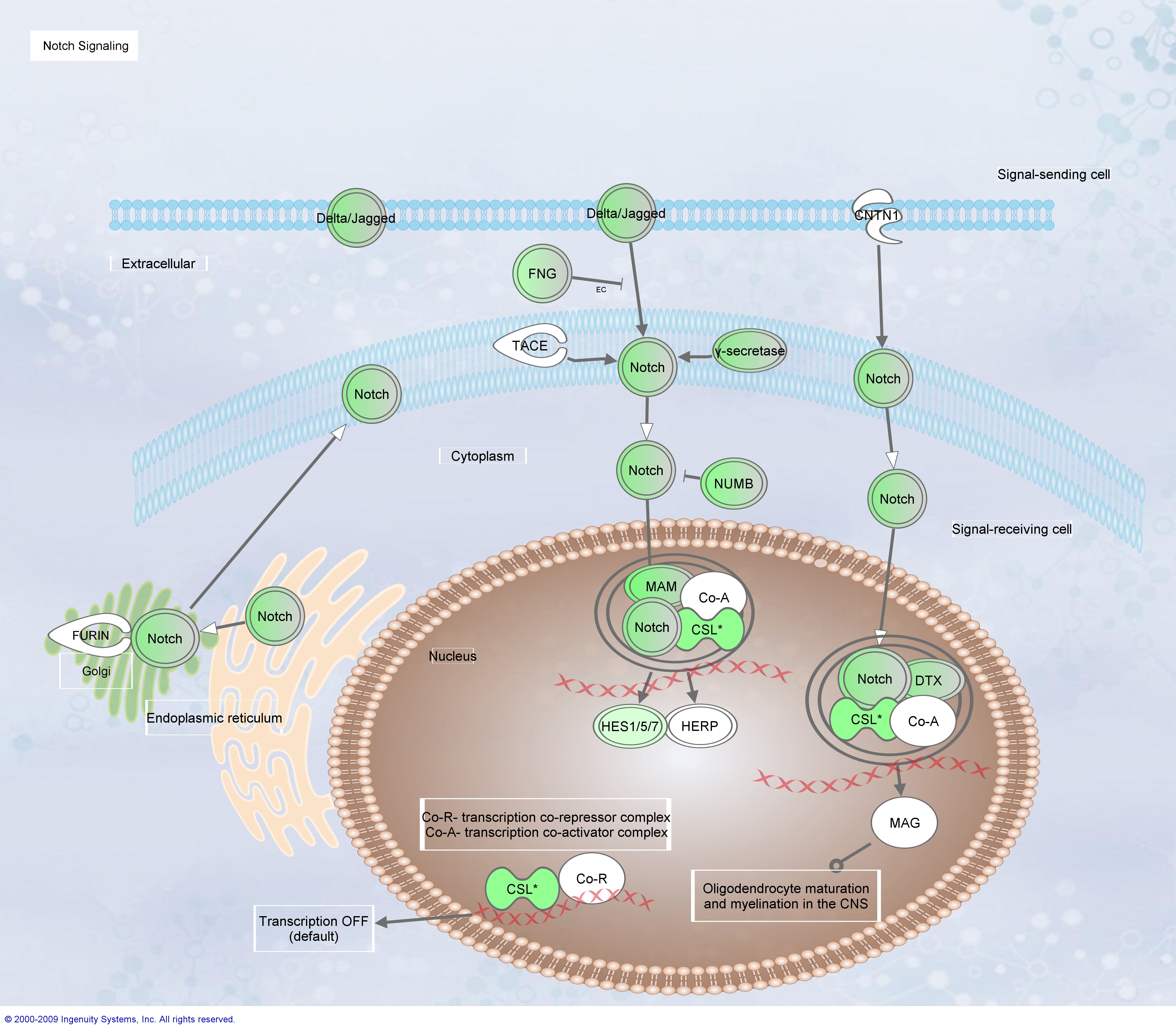

Supplement: Additional file 4 — Figure S2, Notch pathway. Notch canonical pathway. Color corresponds to increase (red) or decrease (green) in signal intensity (expression) of genes in TOF subjects relative to control subjects (developed using the Ingenuity Pathways Analysis program). [file 1755-8794-4-1-S4.JPEG]
